# The Nicotinic Acetylcholine Receptor–Macrophage Axis in Merkel Cell Carcinoma: Evidence, Limitations, and Therapeutic Hypotheses

**DOI:** 10.3390/ijms27146331

**Published:** 2026-07-16

**Authors:** Jeymily Ares-Estrada, Ian García-Quiñones, José A. Lasalde-Dominicci, Leomar Y. Ballester, Phyu P. Aung, Manuel Delgado-Vélez

**Affiliations:** 1 Department of Pharmaceutical Sciences, School of Pharmacy, University of Puerto Rico, San Juan, PR 00936, USA; jeymily.ares@upr.edu; 2 Department of Biology, University of Puerto Rico, San Juan, PR 00925, USA; ian.garcia9@upr.edu (I.G.-Q.); jose.lasalde@upr.edu (J.A.L.-D.); 3 Molecular Sciences Research Center, University of Puerto Rico, San Juan, PR 00936, USA; 4 University of Puerto Rico Comprehensive Cancer Center, San Juan, PR 00936, USA; 5 Division of Pathology and Laboratory Medicine, The University of Texas MD Anderson Cancer Center, Houston, TX 77030, USA; lyballester1@mdanderson.org (L.Y.B.); paung@mdanderson.org (P.P.A.)

**Keywords:** Merkel cell carcinoma, nicotinic acetylcholine receptors, macrophage, skin cancer, inflammation, tumor microenvironment, tumor progression, Merkel cell polyomavirus

## Abstract

Merkel cell carcinoma (MCC) is an aggressive neuroendocrine skin malignancy characterized by high mortality and a rising incidence. Although immune checkpoint inhibitors have significantly improved patient outcomes, many patients exhibit primary or acquired resistance, frequently in the setting of an immunosuppressive tumor microenvironment (TME) enriched for myeloid populations. This review synthesizes current evidence and proposes a testable model in which nicotinic acetylcholine receptor (nAChR) signaling may intersect with tumor-associated macrophage (TAM) biology to reinforce immune exclusion in MCC. Direct MCC-specific evidence currently supports nAChR-subunit expression in tumor tissues and the association of CD163^+^/CD14^+^/S100A8^+^ myeloid populations with resistance to PD-1 pathway blockade; however, functional nAChR signaling in MCC tumor cells, TAMs, or dendritic cells has not yet been demonstrated. We therefore distinguish established MCC observations from mechanistic hypotheses extrapolated from macrophage biology, cholinergic anti-inflammatory signaling, and other cancer models. Within this framework, α7-nAChR signaling on TAMs may activate the cholinergic anti-inflammatory pathway and favor suppressive myeloid states, whereas α3- and α5-containing receptors detected in MCC tumor cells may represent tumor-cell-associated candidates for future mechanistic testing. By integrating evidence on TAM plasticity, spatial immune exclusion, and cholinergic signaling, we propose that the nAChR–macrophage axis is a rational but unproven therapeutic hypothesis that warrants systematic validation in MCC models.

## 1. Introduction

Merkel cell carcinoma (MCC) is a rare but highly aggressive neuroendocrine skin malignancy with a mortality rate significantly higher than that of cutaneous melanoma [1]. The incidence of MCC in the United States grew by 95% between 2000 and 2013, surpassing the growth rates of melanoma (57%) and other solid tumors (15%) [2]. The pathogenesis of MCC is primarily driven by two distinct mechanisms: the clonal integration of the Merkel Cell Polyomavirus (MCPyV) into the host genome [3], which occurs in approximately 80% of cases, or by extensive DNA damage caused by chronic ultraviolet (UV) radiation [4,5]. Furthermore, chronic immunosuppression, such as that seen in organ transplant recipients or patients with HIV, significantly increases the risk of developing MCC, highlighting the critical role of host immunity in controlling this disease [6,7,8].

While MCC is highly immunogenic, it frequently progresses by establishing a complex, immunosuppressive tumor microenvironment (TME). Although the emergence of immune checkpoint inhibitors (ICIs) targeting the PD-1/PD-L1 axis has revolutionized the treatment of advanced MCC, nearly half of all patients do not respond to these therapies or eventually develop resistance [9,10]. Recent evidence suggests that the distribution and phenotype of immune cells within the TME are more predictive of clinical outcomes than the presence of tumor cells alone [11,12,13], underscoring the importance of immune cells in MCC. 

Central to this local immune alteration are tumor-associated macrophages (TAMs), which constitute a major portion of the immune infiltrate in MCC [14]. These cells represent the predominant myeloid population in MCC tumors and exhibit immunosuppressive transcriptional profiles consistent with monocytic myeloid-derived suppressor-like phenotypes [14]. Recent studies have identified subsets of CD163^+^, CD14^+^, and S100A8^+^ TAMs that infiltrate MCC tumors and are associated with resistance to PD-1 pathway blockade, suggesting a role in limiting effective anti-tumor immune responses [14,15]. While macrophage-mediated immunosuppression in other tumor types is frequently associated with an M2-like phenotype characterized by secretion of cytokines such as IL-10 and TGF-β that suppress effector T-cell function and promote angiogenesis [15], the specific cytokine mechanisms and spatial organization of TAM-driven immune regulation in MCC remain incompletely defined.

An emerging area of interest in the MCC TME is the influence of neuro-inflammatory signaling, specifically through nicotinic acetylcholine receptors (nAChRs). To avoid implying that normal Merkel cells are endocrine cells, it is important to distinguish normal Merkel-cell biology from MCC differentiation. Normal Merkel cells are specialized mechanosensory epithelial cells associated with sensory nerve endings; their mechanotransduction depends in part on PIEZO2, and PIEZO2 immunoreactivity has also been reported in MCC tissues [16,17]. At the same time, MCC is classified as a cutaneous neuroendocrine carcinoma because tumor cells commonly express neuroendocrine markers and display neuroendocrine differentiation. Within this context, recent research has identified α3-, α5-, and α7-nAChR subunit expression in MCC tissues [18]. Activation of α7-nAChRs on macrophages suppresses pro-inflammatory cytokine production in non-MCC inflammatory models [19,20,21] and has been associated with M2-like macrophage polarization in other settings [22]. These findings support the hypothesis that nAChR signaling may contribute to immune suppression and an immune-cold or immune-excluded TME in MCC, although this has not yet been directly tested functionally in MCC models.

This review is a hypothesis-generating synthesis of current knowledge regarding systemic inflammation, local myeloid organization, and cholinergic signaling in the MCC tumor microenvironment. We specifically examine the role of TAMs in therapy resistance, the limited but emerging evidence for nAChR expression in MCC, and the experimental steps needed to determine whether nAChR-mediated signaling functionally contributes to macrophage-driven immune exclusion. To improve interpretability, Table 1 separates direct MCC-specific evidence from mechanistic inferences derived from other cancer or inflammatory models.

## 2. Tumor Microenvironment and Inflammation in MCC

Early studies on MCC primarily concentrated on tumor-cell biology, viral oncogenesis driven by MCPyV, and clinicopathologic characteristics [1,3,12]. With the introduction of immunotherapy as an effective treatment strategy for MCC, research has increasingly shifted toward characterizing the immune microenvironment present in MCC tumors [9,10,13,14]. One early immunohistochemical study demonstrated highly heterogeneous infiltration by T lymphocytes and macrophages [29].

**Figure 1:**
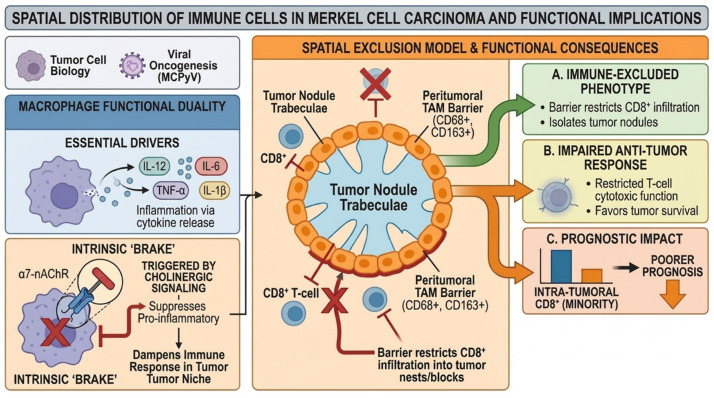
Spatial distribution of immune cells in MCC and proposed functional implications. The schematic shows a TAM-enriched tumor–stroma interface that may restrict CD8^+^ T-cell entry into tumor nests and highlights α7-nAChR as a proposed anti-inflammatory brake on macrophage activation. This nAChR component is hypothesis-generating and requires direct functional validation in MCC.

Rather than treating inflammation as uniformly pro-tumor or anti-tumor, this review distinguishes acute immunostimulatory inflammation, which can support dendritic-cell maturation, antigen presentation, and effector T-cell priming, from chronic suppressive inflammation, in which persistent myeloid recruitment, checkpoint induction, and cholinergic anti-inflammatory signaling may blunt antitumor immunity. This conceptual distinction is central to the model summarized in Figure 1.

### 2.1. Prognostic Significance of Immune Cells and Inflammation in MCC Tumors

In MCC, the immune microenvironment has clear prognostic value, but the cellular markers require explicit interpretation. CD3 marks total T lymphocytes, CD8 marks cytotoxic T cells, CD16 commonly identifies natural killer cells or FcγRIII-expressing immune cells, FoxP3 marks regulatory T cells, and CD68 marks macrophages or monocyte-lineage cells. In a population-based cohort, higher CD3^+^ and CD8^+^ T-cell densities at the tumor periphery were associated with improved overall survival, and higher peripheral CD8^+^ density correlated with improved disease-specific survival. The prognostic effect was strongest in MCPyV-positive tumors, although high CD3^+^ counts were favorable regardless of viral status [13].

MCPyV DNA-positive MCCs have been reported to contain higher densities of CD3^+^, CD8^+^, CD16^+^, FoxP3^+^, and CD68^+^ cells than MCPyV-negative tumors, supporting the idea that viral antigenicity can shape a more inflamed TME [23]. A large European cohort similarly found that CD8^+^ lymphocytes were the strongest single lymphocyte subset associated with overall and disease-specific survival and proposed an immunoscore incorporating CD3, CD8, FoxP3, and PD-L1 [25]. These findings do not mean that all immune markers are uniformly present in only a minority of MCCs. Rather, robust intratumoral CD8^+^ infiltration appears restricted to a subset of tumors, whereas other immune populations vary by viral status, tumor location, and spatial compartment [24]. Thus, immune-cell density, identity, and localization are clinically relevant biomarkers that may help explain prognosis and immunotherapy response.

### 2.2. Spatial Distribution of Immune Cells

Immunohistochemical and spatial studies have consistently identified TAMs as a prominent immune population in MCC tumors [13,29,30]. These studies characterize many MCCs as having an “immune-excluded” phenotype, where TAMs (CD68+, CD163+) are often concentrated at the tumor–stroma interface and at the edges of tumor nodules, effectively isolating tumor trabeculae [13,29,30]. This peritumoral macrophage barrier correlates with decreased infiltration of cytotoxic T cells into tumor nests and a reduced capacity to mount effective antitumor immune responses [13,29] (Figure 1). The strength of these studies lies in their histopathologic and spatial characterization of immune-cell localization; however, small sample size and static tissue analysis limit the functional interpretation of the observed TAM populations [13,29]. This immune-excluded spatial model is summarized in Figure 1.

### 2.3. Local Inflammatory Signals

The inflammatory milieu in MCC is uniquely shaped by the interplay between viral components and host innate immune responses. Infection with the MCPyV has been shown to induce an antiviral innate immune response in primary human dermal fibroblasts, characterized by the activation of the cyclic GMP-AMP synthase–stimulator of interferon genes pathway and the subsequent production of type I interferons (IFNs) and interferon-stimulated genes [31]. However, the virus utilizes mechanisms to evade this host response. Specifically, the MCPyV small T (sT) antigen acts as a potent inhibitor of the NF-κB signaling pathway by targeting the NF-κB essential modulator (NEMO) and recruiting protein phosphatase 4, which deactivates the IKK complex and suppresses pro-inflammatory cytokine production [32,33].

A critical factor in the dissemination and persistence of MCPyV is the role of the myeloid compartment. Research has identified that inflammatory monocytes (CD14^+^CD16^−^) serve as a reservoir for MCPyV, carrying the viral DNA in the peripheral blood [34]. This finding is particularly important, as there is a possibility that TAMs may originate from monocytes that have been altered by MCPyV. This suggests that the immunosuppressive “M2-like” phenotype observed in MCC TAMs may be influenced by viral presence or signaling prior to their recruitment and differentiation at the tumor site.

Despite these mechanistic insights, significant gaps remain in our understanding of the MCC cytokine network. Cytokines such as IL-6 and TNF-α, and chemokine pathways such as CXCL12/CXCR4, are well-documented regulators of myeloid recruitment and tumor progression in other cutaneous and neuroendocrine malignancies [35,36], but their specific expression profiles, cellular sources, and functional roles in the MCC TME remain incompletely characterized. This gap is particularly relevant in light of the findings by Cunningham et al. (2025), who identified α3-, α5-, and α7-nAChR subunit expression in human MCC tissues, with α5 and α7 detected in most cases [18]. In non-MCC macrophage models, α7-nAChR activation can suppress pro-inflammatory cytokines such as IL-6 and TNF-α [19]. Therefore, nAChR-mediated signaling could plausibly act as a local inflammatory “brake” that contributes to immune exclusion or cytokine remodeling in MCC, but this possibility remains unproven. Determining whether cholinergic signaling influences monocyte recruitment, TAM differentiation, or peritumoral barrier formation will require MCC-specific functional studies.

### 2.4. MCPyV-Positive Versus MCPyV-Negative MCC: Implications for TME, TAMs, and nAChR Hypotheses

MCPyV-positive and MCPyV-negative MCC represent biologically distinct routes to a similar neuroendocrine carcinoma phenotype. MCPyV-positive tumors are driven by clonal viral integration and continued expression of viral T antigens, whereas MCPyV-negative tumors usually show UV-mutational signatures and higher mutational burden [3,4,5]. These etiologic differences influence antigenicity: MCPyV-positive tumors can present viral oncoprotein-derived antigens, whereas MCPyV-negative tumors may generate UV-associated neoantigens. Clinically and epidemiologically, both subtypes occur predominantly in older and immunologically vulnerable patients, but virus-negative disease is often more closely linked to chronic UV exposure and can show more genomically damaged molecular profiles [2,4,5].

The TME also differs by viral status. MCPyV-positive tumors have been associated with higher densities of CD3^+^, CD8^+^, CD16^+^, FoxP3^+^, and CD68^+^ immune cells than MCPyV-negative tumors, and MCPyV status has been incorporated into prognostic immune models [13,23,25]. However, the relationship between viral status and TAM biology is not yet resolved. Available MCC studies support the relevance of CD163^+^/CD14^+^/S100A8^+^ myeloid populations in ICI resistance [14], but they do not yet define whether TAM recruitment, localization, cytokine output, or nAChR expression differs reproducibly between MCPyV-positive and MCPyV-negative tumors. This is a priority for future spatial and functional studies.

The limited MCC-specific nAChR data also suggest viral-subtype relevance. In the current MCC tissue study, MCPyV-positive tumors were more likely to express nuclear/cytoplasmic α3-nAChR and α5-nAChR, and high-intensity α5-nAChR staining correlated with viral positivity [18]. These associations do not establish functional receptor signaling, but they provide a rationale to stratify future nAChR studies by MCPyV status. In particular, mechanistic experiments should test whether α3/α5-containing receptor complexes are preferentially assembled or functional in MCPyV-positive tumor cells, and whether α7-nAChR expression or activity in TAMs differs by viral subtype.

## 3. TAMs in MCC

TAMs are the primary orchestrators of immunosuppression in MCC. While early research focused on density as a prognostic marker, high-dimensional profiling (scRNA-seq, CODEX) has revealed that their monocytic myeloid-derived suppressor cell-like functional state and spatial localization at the tumor–stroma interface are the true drivers of progression and immunotherapy resistance [14]. This spatial architecture predicts treatment failure more reliably than traditional density measurements [14].

### 3.1. Prevalence and Markers of TAMs in MCC

TAMs, typically identified as CD68^+^ cells, are abundant in nearly all primary MCC specimens. Single-cell RNA sequencing (scRNA-seq) has confirmed that macrophages are the dominant myeloid population in MCC tumors, often significantly outnumbering infiltrating T cells. Beyond the pan-macrophage marker CD68, common markers used to define these populations include CD163, a scavenger receptor, and CD14, which is associated with monocyte-derived macrophages. Furthermore, S100A8/A9 (calprotectin) has emerged as a critical marker identifying a subset of myeloid cells with potent immunosuppressive properties and high relevance to treatment failure [14].

### 3.2. TAM Phenotype and Function

In MCC, TAMs play a pivotal role in orchestrating an “immune-excluded” TME. Studies consistently show that these cells predominantly exhibit an M2-like phenotype, characterized by the expression of CD163+ and S100A8/A9+, which correlates with poorer clinical outcomes [14]. Crucially, these macrophages, rather than the tumor cells themselves, are often the primary carriers of immune checkpoint molecules, expressing high levels of PD-L1 and inhibitory receptors from the leukocyte immunoglobulin-like receptor B (LILRB) family. This immunosuppressive program is reinforced by the CD200-CD200R axis, where MCC cells express CD200 to engage receptors on myeloid cells [30], and chemokine signaling such as the CXCL12/CXCR4 axis [29], which may trap these cells at the stroma–tumor interface. 

### 3.3. Impact of TAMs on Therapy

**Figure 2:**
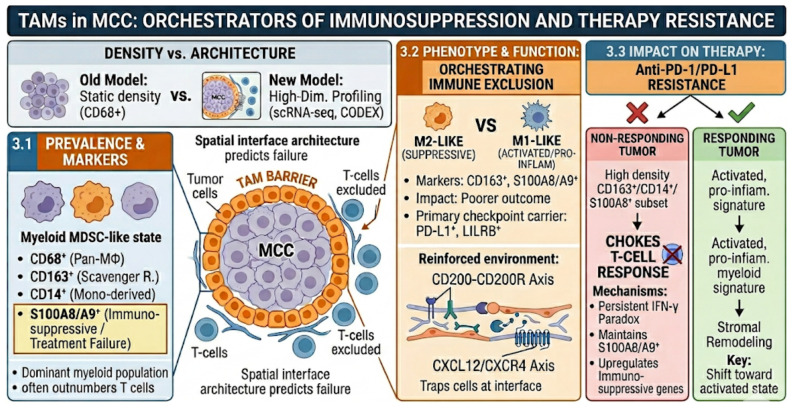
TAM-mediated immune exclusion and therapy resistance in MCC. The schematic emphasizes MCC-associated CD163^+^/CD14^+^/S100A8^+^ myeloid populations, their localization at the tumor–stroma interface, and proposed checkpoint or chemokine pathways that may limit effective T-cell infiltration and contribute to PD-1/PD-L1 resistance.

High prevalence of CD163^+^/CD14^+^/S100A8^+^ myeloid populations has been associated with resistance to anti-PD-1/PD-L1 therapies in MCC, with non-responding tumors often displaying abundant macrophage infiltration that spatially constrains productive T-cell responses [14,15]. Mechanistically, responding tumors typically show signs of stromal remodeling and a shift toward activated, pro-inflammatory myeloid signatures [10]. Conversely, non-responding tumors often exhibit persistent IFN-γ-driven inflammation which, in the MCC environment, paradoxically drives the upregulation of immunosuppressive genes and maintains the S100A8/A9^+^ macrophage population that thwarts PD-1 blockade [14] (Figure 2). Despite these advances, the lack of standardized classification for these macrophage subsets remains a limitation, highlighting the need for functional assays to supplement static immunohistochemical data. These relationships are summarized in Figure 2.

## 4. Inflammation–Macrophage Interactions and MCC Progression

MCC is a highly immunogenic tumor, yet it frequently evades effective immune control [14,37,38,39]. Growing evidence from MCC and other inflammation-driven cancers highlights TAMs as key regulators that interpret and shape inflammatory signals, acting as a molecular switch that determines whether tumor progression is promoted or restrained [14,40,41,42,43,44]. In MCC, the dynamic interaction between macrophages and tumor cells within the inflammatory microenvironment plays a central role in both disease progression and response to immunotherapy [14,15,40,42].

Importantly, the spatial organization and functional state of TAMs dictate their impact on the TME. While TAMs possess the inherent plasticity to support effective CD8+ T-cell-mediated immunity, they more commonly adopt an immunosuppressive phenotype in the MCC environment. In this state, they form specialized peritumoral niches (architectural barriers) that promote tumor growth, facilitate metastasis, and drive resistance to PD-1-based therapies [11,14,40]. 

The proposed model therefore does not imply that inflammation is harmful in MCC. Acute, spatially productive inflammation may promote antigen presentation and recruitment of functional cytotoxic lymphocytes, whereas chronic or unresolved inflammation can select for compensatory checkpoints, suppressive cytokines, myeloid-derived suppressor-like programs, and physical immune exclusion. In this context, cholinergic signaling is hypothesized to act as a negative-feedback circuit that becomes maladaptive when it stabilizes suppressive TAM states instead of resolving inflammation while preserving antitumor immunity.

### 4.1. Immune Evasion

MCC tumors can exploit inflammatory pathways to facilitate immune evasion, primarily through the PD-L1 axis [45]. Additional inhibitory checkpoints beyond PD-L1, including TIGIT, have also been reported in MCC, emphasizing that immune escape is not limited to a single checkpoint axis [46]. This does not contradict the favorable prognostic association of CD8^+^ T-cell infiltration. Rather, it reflects adaptive immune resistance: CD8^+^ T cells and other activated immune cells can produce IFN-γ, and IFN-γ can induce PD-L1, HLA-A/B/C, IDO1, and other immunoregulatory programs in tumor and stromal compartments [45,47,48]. Thus, high PD-L1 expression in a CD8^+^ T-cell-infiltrated tumor may indicate that antitumor immunity has been recognized by the tumor, but is being restrained by feedback checkpoint pathways. Beyond tumor-intrinsic mechanisms, myeloid cells within the TME, specifically suppressive or M2-like TAMs, may serve as sources of PD-L1 and LILRB-family inhibitory signals, reinforcing local immunosuppression. In this architectural niche, TAM-associated PD-L1 and LILRB-family signaling could amplify T-cell exhaustion and macrophage dysfunction. This evasive state may be further compounded by the downregulation of the major histocompatibility complex class I (MHC I) and by inflammation-suppressive cytokines such as TGF-β and IL-10, although the cellular sources and functional dominance of these cytokines remain incompletely defined in MCC [45,48].

While direct evidence for the macrophage–LILRB axis in MCC is currently being established, strong data from other human solid tumors validate these proposed pathways [49]. TAMs in human cancers typically express PD-L1 and other checkpoint molecules, enabling them to directly suppress T cells while adopting an M2-like polarization [50,51]. Notably, PD-L1 blockade can reprogram these cells toward a tumor-protective M1-like phenotype and enhance their phagocytic activity [50,51]. Furthermore, TAMs exploit “do not eat me” mechanisms, including the LILRB1–MHC I/β2-microglobulin interaction, which directly inhibits macrophage phagocytosis and promotes tumor immune avoidance [49]. Comprehensive reviews of TAM biology underscore that signaling through PD-L1 and LILRB family receptors positions macrophages as central organizers of the immunosuppressive, inflammatory TMEs that drive resistance to PD-1/PD-L1 blockade [49,52,53].

Although this literature is not MCC-specific, studies in testicular germ-cell tumors provide an instructive example of how PD-L1-expressing TAMs can represent a biologically meaningful TME compartment. Melotti et al. reported that CD68/PD-L1 double-positive TAMs vary across germ-cell tumor components during tumor-cell reprogramming, supporting the broader concept that PD-L1-expressing macrophages may participate in tumor-state regulation rather than serving only as passive inflammatory markers [54].

### 4.2. Cytokine Environment

**Figure 3:**
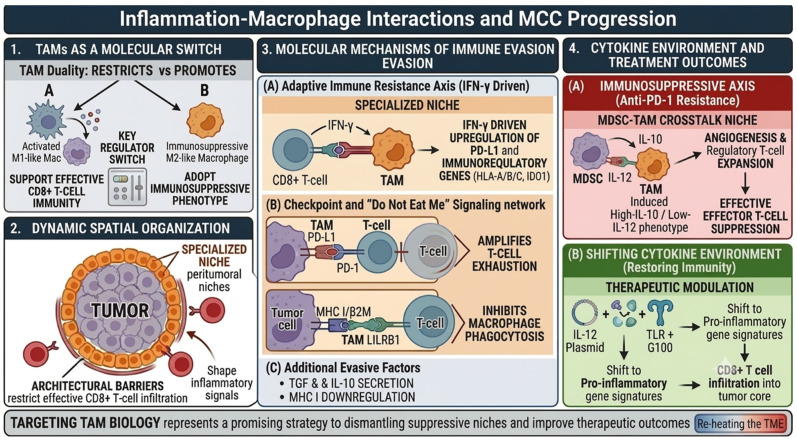
Inflammation- and TAM-mediated immunosuppression in MCC. The schematic contrasts potentially productive antitumor inflammation with chronic suppressive myeloid programs, including PD-L1/LILRB signaling, reduced phagocytosis, and an IL-10/IL-12 imbalance that may limit cytotoxic T-cell function.

The cytokine environment in MCC remains insufficiently defined and should be framed as a knowledge gap rather than as an established suppressive program. In other malignancies, MDSC–TAM crosstalk can promote an immunosuppressive axis in which MDSC-derived IL-10 downregulates MHC II on macrophages, inducing a high-IL-10/low-IL-12 phenotype that supports angiogenesis, regulatory T-cell expansion, and suppression of effector T-cell activity [55]. In MCC, resistance to PD-1/PD-L1 blockade is associated with suppressive myeloid populations and anti-inflammatory programs, but individual TAM-derived IL-10 or TGF-β levels in human MCC tissues have not been systematically reported [48]. Conversely, interventions that modulate the cytokine environment, such as intratumoral IL-12 plasmid approaches or the TLR4 agonist G100, have induced pro-inflammatory gene signatures and CD8^+^ T-cell infiltration, supporting the concept that shifting the cytokine milieu can restore antitumor immunity in selected patients [26,48] (Figure 3). The inflammatory switch model is summarized in Figure 3.

## 5. Targeting Macrophages and Inflammation in MCC

Direct macrophage-targeting trials in MCC remain limited, so therapeutic discussion should be anchored to MCC-specific observations rather than broad TAM biology alone. The most relevant MCC evidence includes the association of CD163^+^/CD14^+^/S100A8^+^ suppressive or M2-like myeloid populations with resistance to PD-1 pathway blockade, the presence of CD200-CD200R and CXCL12/CXCR4 pathways that may support myeloid retention or immune exclusion, and clinical evidence that intratumoral TLR4 agonism can induce inflammatory gene signatures and CD8^+^ T-cell infiltration [14,26,29,30]. Thus, macrophage-directed approaches are best presented as strategies to test and remodel MCC-specific immune exclusion rather than as established therapies for MCC.

Accordingly, macrophage-directed approaches in MCC can be prioritized into four experimentally testable categories: reprogramming suppressive or M2-like TAMs with TLR, CD40, or PI3Kγ-directed strategies; disrupting recruitment or retention pathways such as CCL2/CCR2, CSF1/CSF1R, or CXCL12/CXCR4; blocking myeloid checkpoints such as CD47-SIRPα, LILRB-family receptors, TREM2, or VISTA; and combining these approaches with PD-1/PD-L1 blockade in ICI-resistant disease. Depletion strategies should be interpreted cautiously, because macrophages can also support antigen presentation, tissue remodeling, and productive antitumor immunity depending on context.

### Macrophage-Targeted Approaches

**Figure 4:**
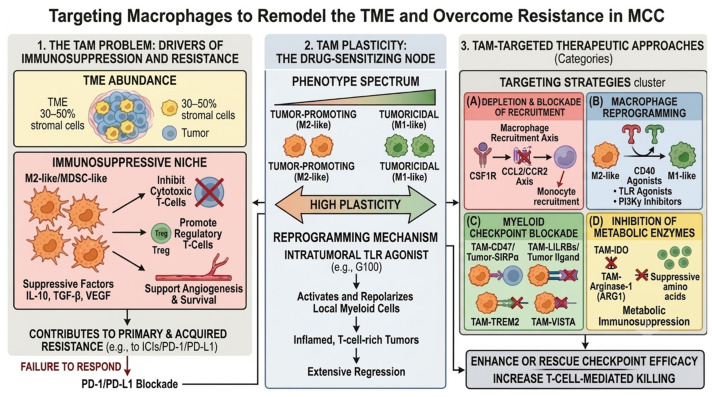
Therapeutic strategies targeting the macrophage compartment in MCC. The schematic groups potential approaches into myeloid reprogramming, recruitment or retention blockade, myeloid checkpoint inhibition, and metabolic modulation, with emphasis on combination strategies designed to overcome immune exclusion and improve checkpoint blockade.

Within this MCC-focused framework, broad TAM-targeted drug classes are discussed as candidate tools for testing specific MCC mechanisms rather than as generic therapeutic claims. CSF1R or CCL2/CCR2 blockade would test whether monocyte recruitment sustains the peritumoral macrophage barrier; CXCL12/CXCR4 inhibition would test stromal retention and immune exclusion; CD40 or TLR agonism would test whether suppressive TAMs can be converted into antigen-presenting, T-cell-supportive states; and CD47-SIRPα, LILRB-family, TREM2, or VISTA blockade would test whether myeloid checkpoint signaling limits phagocytosis and T-cell activation. These approaches should be evaluated with MCC-relevant endpoints, including CD8^+^ intratumoral penetration, TAM spatial redistribution, PD-L1/LILRB expression, MCPyV/UV subtype, and response to PD-1/PD-L1 blockade (Figure 4). These experimental categories are consistent with the broader literature on TAM-targeting in solid tumors [56,57,58,59,60,61,62,63,64,65,66,67,68,69,70,71,72].

## 6. nAChR Signaling in MCC: Direct Evidence, Ligand Sources, and Functional Hypotheses

The strongest direct MCC-specific evidence for cholinergic receptor involvement is tissue-level detection of nAChR subunits. In an immunohistochemical survey of 71 primary human MCCs, tumor cells frequently expressed α3-, α5-, and α7-nAChR subunits. MCPyV-positive tumors were more likely to express nuclear/cytoplasmic α3-nAChR and α5-nAChR than virus-negative cases, high-intensity α5-nAChR staining correlated with viral positivity, and α7-nAChR localization was associated with infiltrative growth patterns [18]. These findings extend an older series in which all 15 human MCCs examined were diffusely positive for muscarinic M3/M5 acetylcholine receptors but lacked the β2-nAChR subunit [73].

Preliminary unpublished confocal imaging observations from our group further suggest that nAChR immunoreactivity can be accentuated within tumor-cell-rich regions rather than predominantly peritumoral areas in both MCPyV-positive and MCPyV-negative MCC specimens. Because these observations have not yet undergone peer review and do not establish receptor assembly, cell-surface localization, ligand responsiveness, or downstream signaling, we treat them as hypothesis-generating spatial evidence that strengthens the rationale for formal compartment-resolved validation rather than as proof of a functional cholinergic mechanism.

**Figure 5:**
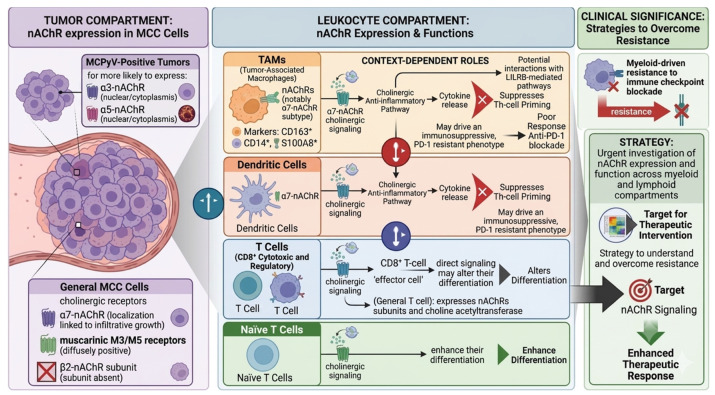
Context-dependent roles of cholinergic signaling in the MCC microenvironment. The schematic distinguishes direct MCC evidence for tumor-cell nAChR-subunit expression from hypothesized effects on TAMs, dendritic cells, and T cells based on broader cholinergic immunology. Functional validation is needed to determine whether these interactions contribute to PD-1/PD-L1 resistance.

The MCC tumor microenvironment contains immune populations that can express nAChRs in other biological contexts, including TAMs, dendritic cells, cytotoxic T cells, and regulatory T cells [74,75,76,77,78,79]. In MCC specifically, analyses of pre-immunotherapy tumor samples identified CD163^+^, CD14^+^, and S100A8^+^ TAM subsets associated with resistance to PD-1 blockade and myeloid transcriptional programs enriched for cytokine signaling and LILRB-family receptors [14]. These observations support, but do not prove, a model in which cholinergic signaling could modulate myeloid-driven immunosuppression in MCC (Figure 5).

### 6.1. Direct Evidence and Current Limitations

At present, nAChR expression in MCC should not be equated with functional receptor signaling. Immunohistochemistry or transcript detection does not establish receptor assembly into functional pentamers, cell-surface localization, ligand accessibility, ion-channel activity, ionotropic/metabotropic signaling, or causality for TAM polarization, T-cell exclusion, or ICI resistance. The proposed nAChR-TAM axis should therefore be interpreted as a biologically plausible and experimentally testable mechanism, not as an established driver of MCC progression. This question is also currently under investigation by our group.

### 6.2. Potential Sources of Acetylcholine or Cholinergic Agonists in the MCC TME

A critical unresolved issue is ligand availability. Potential sources of acetylcholine or nAChR agonists within the MCC microenvironment include autonomic or sensory nerve fibers, keratinocytes and other non-neuronal skin cells, endothelial or stromal cells, ChAT-expressing immune cells, and possibly MCC tumor cells themselves [76,77,78,80,81,82]. Exogenous nicotine or tobacco-derived nitrosamines could also activate nAChRs, although their relevance to MCC has not been established. No study has yet mapped acetylcholine, choline acetyltransferase, vesicular acetylcholine transporter, acetylcholinesterase, butyrylcholinesterase, or organic cation transporter expression spatially in MCC tissue. Defining ligand sources is therefore essential before assigning functional importance to nAChR expression in tumor cells or TAMs. This is a matter that we are also currently exploring.

### 6.3. Effects of nAChRs on Inflammation and Tumor Progression

**Figure 6:**
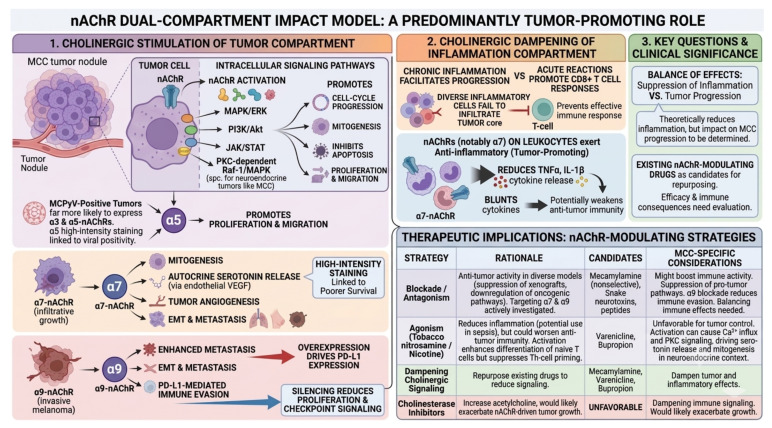
Proposed dual-compartment impact of nAChR signaling in MCC. The schematic summarizes hypothesized tumor-cell effects, immune-cell effects, and therapeutic considerations for nAChR modulation. The model is derived largely from non-MCC systems and should be tested directly in MCC.

Activation of nAChRs can influence tumor growth and immune responses in several non-MCC systems, especially models related to nicotine or tobacco-derived nitrosamine exposure. In these settings, nAChR signaling has been associated with proliferation, survival, migration, angiogenesis, and activation of MAPK/ERK, PI3K/Akt, JAK/STAT, and related pathways [83,84,85,86,87,88,89]. For example, in small-cell lung cancer, a neuroendocrine carcinoma used here only as an analogy, α7-nAChR activation by a tobacco nitrosamine induced calcium influx and PKC-dependent Raf-1/MAPK signaling, promoting autocrine serotonin release and mitogenesis [83,90]. Whether similar signaling occurs in MCC is unknown. Therefore, cholinergic stimulation of MCC cells should be considered a testable possibility rather than an established mechanism (Figure 6).

Evidence from melanoma and other cancers further illustrates why nAChRs may be worth investigating in MCC, but these data should not be treated as MCC-specific proof. In melanoma, α5-nAChR has been linked to proliferation and migration through AKT/Notch1 signaling [91], and α9-nAChR has been associated with invasive behavior, metastasis, and PD-L1-mediated immune evasion [92,93]. These examples support the broader concept that nAChR signaling can influence tumor-cell behavior and immune-evasion programs in some cancers. For MCC, however, they justify mechanistic experiments rather than therapeutic conclusions.

This model also requires explicit reconciliation of inflammatory timing and function. Acute inflammatory activation can promote dendritic-cell maturation, antigen presentation, and productive antitumor T-cell responses, whereas chronic inflammation can sustain suppressive myeloid recruitment, checkpoint expression, angiogenesis, and tissue remodeling [94]. In MCC, inflammatory cells often accumulate at tumor margins but may fail to penetrate tumor nests effectively [29]. α7-nAChR activation on these immune populations could theoretically reduce damaging inflammation, but in a tumor context it may also stabilize an immune-excluded niche; the direction and magnitude of this effect remain unknown in MCC.

On the immunomodulatory side, α7-nAChR signaling on macrophages and monocytes is best supported as an anti-inflammatory pathway that blunts TNF-α, IL-1β, and related cytokine release [19,20]. Whether this is tumor-promoting in MCC depends on context: it could limit harmful chronic inflammation, but it could also weaken antigen presentation, T-cell priming, and cytokine release needed for durable antitumor immunity. In the MCC cohort reported by Cunningham et al. [18], high-intensity α7-nAChR staining was associated with poorer overall survival in univariable survival analysis, but this association did not remain significant after multivariable adjustment. Thus, the finding is consistent with, but does not prove, a suppressive or tumor-promoting role for α7-nAChR signaling in MCC [18].

In summary, most mechanistic evidence for tumor-promoting nAChR activity comes from non-MCC systems, where nAChR activation has been linked to proliferation, angiogenesis, metastasis, and immune evasion. For MCC, the appropriate conclusion is narrower: nAChR subunits are present in tumor tissues, and their functional significance should be tested directly using genetic, pharmacological, biochemical, and electrophysiological approaches. No tumor-suppressive or tumor-promoting nAChR function has yet been established in MCC.

### 6.4. Therapeutic Implications

Because nAChR-targeted therapies have not been tested clinically or preclinically in MCC, therapeutic implications should be prioritized according to the limited MCC-specific evidence. The most mechanistically compelling immune-cell target is α7-nAChR in TAMs and dendritic cells, because this receptor is linked to cholinergic anti-inflammatory signaling and could plausibly regulate cytokine output, antigen presentation, and T-cell exclusion. Tumor-cell-associated α5- and α3-containing receptors are also priorities because α5 and α3 expression patterns in MCC tissues correlate with MCPyV status and histopathologic parameters [18]. By contrast, α9-nAChR is best regarded as a lower-priority extrapolated candidate, supported mainly by melanoma and breast cancer models rather than MCC data [92,93,95,96]. Before considering repurposing agents such as mecamylamine, varenicline, bupropion, cholinesterase-modulating agents, or α7/α9-selective ligands for MCC [27,28,95,96,97,98], studies should validate receptor assembly, cell-surface localization, ligand availability, receptor function, and effects on TAM polarization, tumor-cell signaling, cytokine release, T-cell infiltration, and response to PD-1/PD-L1 blockade. Thus, nAChR modulation is best framed as a preclinical research direction rather than an immediately actionable therapeutic strategy.

## 7. Conclusions

MCC progression and immunotherapy resistance are shaped by tumor-intrinsic viral or UV-driven biology and by the spatial organization of the immune microenvironment. The current evidence supports TAM-mediated immune exclusion as an important MCC-relevant process, while nAChR involvement remains a plausible but unproven hypothesis. Direct MCC data demonstrate nAChR-subunit expression in tumor tissues, but not functional receptor signaling in tumor cells or TAMs. Future studies should therefore test whether cholinergic signaling stabilizes suppressive myeloid states, contributes to T-cell exclusion, or can be therapeutically modulated to improve checkpoint blockade.

## 8. Key Unanswered Questions and Prioritized Research Roadmap

Several questions should be prioritized. First, spatial multi-omics studies should map α3-, α5-, α7-, and other nAChR subunits together with TAM markers, T-cell localization, MCPyV status, PD-L1/LILRB expression, and clinical response to ICIs. Second, ligand-source studies should define whether acetylcholine is supplied by nerve fibers, keratinocytes, immune cells, stromal cells, tumor cells, or exogenous nicotine exposure in MCC tissues. Third, receptor function should be validated using orthogonal approaches, including subunit knockdown or knockout, genetic rescue, receptor-complex analysis, co-immunoprecipitation or proximity assays, cell-surface biotinylation, ligand-binding assays, calcium imaging, electrophysiology, and pharmacological agonism/antagonism. Fourth, macrophage-focused assays should determine whether α7-nAChR activity alters TAM cytokine output, antigen-presentation capacity, phagocytosis, LILRB/PD-L1 expression, and T-cell exclusion. Finally, organotypic cultures, patient-derived models, or immune-competent experimental systems should test whether nAChR modulation enhances PD-1/PD-L1 blockade, TLR agonism, or other myeloid-reprogramming strategies. This roadmap would convert the present hypothesis from a conceptual framework into a set of experimentally verifiable mechanisms.

## Figures and Tables

**Table 1:** Evidence hierarchy supporting the proposed nAChR–macrophage axis in Merkel cell carcinoma.

Evidence	Direct Support Relevant to Merkel Cell Carcinoma	Limitations for the Nicotinic Acetylcholine Receptor–Tumor-Associated Macrophage Model
Established MCC biology	MCC arises through MCPyV-associated oncogenesis or UV-associated mutagenesis, and immune infiltration and ICI resistance are clinically relevant [2,3,4,5,9,10,11,12,13].	These observations establish immunogenicity and resistance but do not identify cholinergic signaling as a causal mechanism.
MCC-specific immune/TME evidence	CD8^+^ T-cell density and spatial distribution have prognostic value and CD163^+^/CD14^+^/S100A8^+^ myeloid populations are associated with resistance to PD-1 pathway blockade [14,23,24,25].	TAM phenotype, cytokine output, and spatial function remain incompletely standardized across cohorts and viral subtypes.
Direct nAChR evidence in MCC	Human MCC tissues express α3-, α5-, and α7-nAChR subunits; α3/α5 patterns are enriched in MCPyV-positive tumors, and α7-nAChR localization has been linked to infiltrative growth, while its intensity has been associated with worse overall survival [18].	Expression by IHC does not prove receptor assembly, ligand availability, ion-channel activity, or biological causality.
Mechanistic inference from non-MCC systems	α7-nAChR signaling can suppress macrophage cytokine production and influence macrophage polarization in inflammatory models [19,20,22].	These findings support a plausible mechanism but cannot be assumed to operate in MCC-associated TAMs without functional validation.
Therapeutic hypothesis	Myeloid reprogramming and TLR agonism can reshape immune responses in MCC, and nAChR-modulating drugs exist for other indications [26,27,28].	No nAChR agonist or antagonist has yet been tested as an MCC therapy; clinical relevance remains preclinical and speculative.

## Data Availability

The original contributions presented in this study are included in the article. Further inquiries can be directed to the corresponding author.

## Abbreviations

The following abbreviations are used in this manuscript:

MCC	Merkel cell carcinoma
MCPyV	Merkel cell polyomavirus
TME	Tumor microenvironment
ICIs	Immune checkpoint inhibitors
PD-1	Programmed cell death protein 1
PD-L1	Programmed death-ligand 1
TAMs	Tumor-associated macrophages
nAChRs	Nicotinic acetylcholine receptors
scRNA-seq	Single-cell RNA sequencing
CODEX	CO-Detection by indexing
MDSCs	Myeloid-derived suppressor cells
LILRB	Leukocyte immunoglobulin-like receptor B family
IFN-γ	Interferon-gamma
HLA-A/B/C	Human leukocyte antigen A/B/C
IDO1	Indoleamine 2,3-dioxygenase 1
MHC I	Major histocompatibility complex class I
TGF-β	Transforming growth factor beta
IL-10	Interleukin 10
IL-12	Interleukin 12
MHC II	Major histocompatibility complex class II
TLR	Toll-like receptor
CSF1R	Colony-stimulating factor 1 receptor
CCL2	C-C Motif Chemokine Ligand 2
CCR2	C-C Motif Chemokine Receptor 2
PI3Kγ	Phosphoinositide 3-kinase gamma
SIRPα	Signal regulatory protein alpha
TREM2	Triggering receptor expressed on myeloid cells 2
VISTA	V-domain Ig suppressor of T cell activation
ARG1	Arginase-1
sT antigen	Small T antigen
NEMO	NF-κB essential modulator
IKK complex	IκB kinase complex
MAPK/ERK	Mitogen-activated protein kinase/extracellular signal-regulated kinase
PI3K/Akt	Phosphoinositide 3-kinase/protein kinase B
JAK/STAT	Janus kinase/signal transducer and activator of transcription
